# Contralateral Occult Inguinal Hernia Unmasked During Final Low-Pressure Inspection in a Transabdominal Preperitoneal (TAPP) Procedure: A Case Report

**DOI:** 10.7759/cureus.91551

**Published:** 2025-09-03

**Authors:** Yoichi Miyaoka, Shingo Shimada, Tomoya Saito, Shota Ebinuma, Ryoji Yokoyama

**Affiliations:** 1 General Surgery, Abashiri-Kosei General Hospital, Abashiri, JPN; 2 General Surgery, Otaru General Hospital, Otaru, JPN

**Keywords:** contralateral hernia, laparo-endoscopic repair, low-pressure inspection, myopectineal orifice, occult inguinal hernia, transabdominal preperitoneal repair

## Abstract

Transabdominal preperitoneal (TAPP) repair allows assessment of both groins, yet small contralateral defects may be pressure-dependent and inapparent at the initial survey. We report a case of an 87-year-old patient with a reducible left groin bulge. At the start of TAPP, intraperitoneal inspection showed a left direct inguinal hernia, classified as M3 according to the European Hernia Society (EHS) classification, and no obvious right-sided defect. After left-sided preperitoneal dissection, mesh placement, and peritoneal closure, the pneumoperitoneum - initially maintained at 10 mmHg - was reduced to 7 mmHg for a final bilateral inspection before trocar removal. At that point, a subtle bulge at the right internal ring, corresponding to an indirect inguinal hernia (L1 in the EHS classification), became evident and was repaired in the same session with preperitoneal mesh and peritoneal closure. The operative time was 93 minutes, blood loss was negligible, and recovery was uneventful. This case highlights that inspection should not only be performed at the beginning of TAPP but also at the end before closure. A brief low-pressure final check can unmask contralateral occult inguinal hernias that are not visible at the initial high-pressure survey, which might allow for definitive single-session management.

## Introduction

Inguinal hernia repair is one of the most common procedures in general surgery. The transabdominal preperitoneal (TAPP) approach provides excellent visualization of the entire myopectineal orifice, allowing not only repair of the symptomatic side but also assessment of the contralateral groin. Previous studies have demonstrated that a proportion of patients harbor occult contralateral hernias [1-4].

Although international guidelines highlight that laparo-endoscopic repair can detect and treat occult defects [2,5], the optimal strategy for contralateral inspection remains under discussion. Most surgeons perform an initial inspection at the beginning of the procedure; however, the value of conducting a final check before closure has not been emphasized.

We report a case in which a contralateral occult inguinal hernia was not evident at the start of TAPP but was detected during a final low-pressure inspection prior to closure, underscoring the importance of assessing both groins not only at the beginning but also at the end of the procedure.

To aid interpretation, we have used the European Hernia Society (EHS) groin hernia classification throughout this report: “M” denotes direct inguinal defects within Hesselbach’s triangle, “L” denotes indirect defects at the internal ring, and “F” denotes femoral defects; numeric grades (1-3) indicate increasing defect size/extent [2]. This taxonomy - endorsed by international guidelines - helps standardize reporting and guides intraoperative decisions on mesh coverage of the myopectineal orifice and recurrence risk [2,5].

## Case presentation

An 87-year-old man was referred to our department after a day-care medical examination noted a bulge in the left groin. The swelling was reducible and more prominent on standing. The patient denied pain, bowel obstruction symptoms, or previous groin surgery. Daily activities were largely unaffected; however, prolonged standing accentuated the protrusion. There were no recent episodes suggestive of incarceration, nausea, vomiting, or changes in bowel habits. On physical examination, a left inguinal swelling was visible on standing and with gentle Valsalva and became inapparent in the supine position with manual reduction. No palpable or visible defect was appreciated on the right side in either position.

The patient had multiple comorbidities, including prior aortic valve replacement and coronary artery bypass grafting, chronic heart failure with preserved ejection fraction, stage 3b chronic kidney disease, hypertension, dyslipidemia, and type 2 diabetes mellitus.

**Figure 1:**
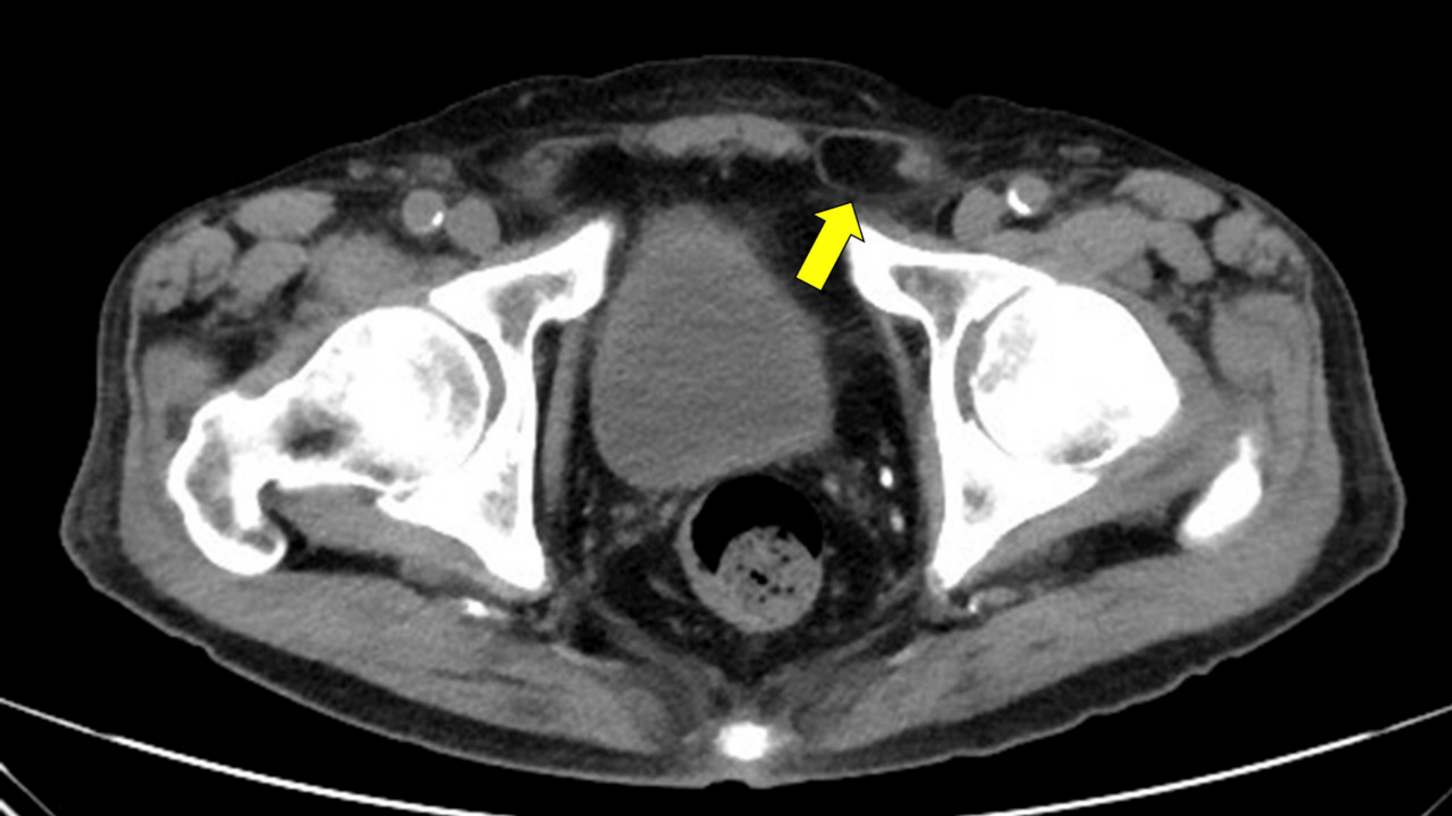
Preoperative computed tomography scan of a left inguinal hernia Preoperative abdominal computed tomography demonstrating a left inguinal hernia (yellow arrow) with protrusion of intra-abdominal fat into the inguinal canal.

Preoperative abdominal computed tomography demonstrated protrusion of intra-abdominal fat into the left inguinal region (Figure 1).

**Figure 2:**
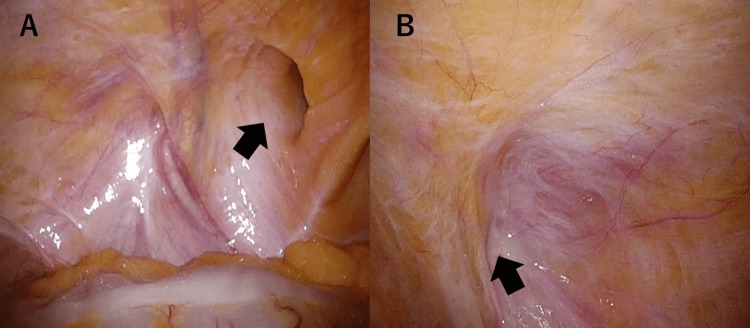
Intraoperative laparoscopic findings at the beginning of the TAPP procedure (A) Left direct inguinal hernia (EHS classification M3) is observed (black arrow). (B) No hernia defect is visible on the right side (black arrow). TAPP: Transabdominal preperitoneal; EHS: European Hernia Society

Under general anesthesia, a standard three-port TAPP repair was performed with an initial pneumoperitoneum pressure of 10 mmHg. At the beginning of the operation, intraperitoneal inspection showed a left direct inguinal hernia, classified as M3 according to the EHS classification, and no evidence of a right-sided defect (Figure 2).

A peritoneal flap was created on the left side, the hernia sac was reduced, and the preperitoneal space was fully exposed with identification of anatomical landmarks. A 10 × 16 cm polypropylene mesh was placed to cover the entire myopectineal orifice, and the peritoneum was closed with a running suture.

**Figure 3:**
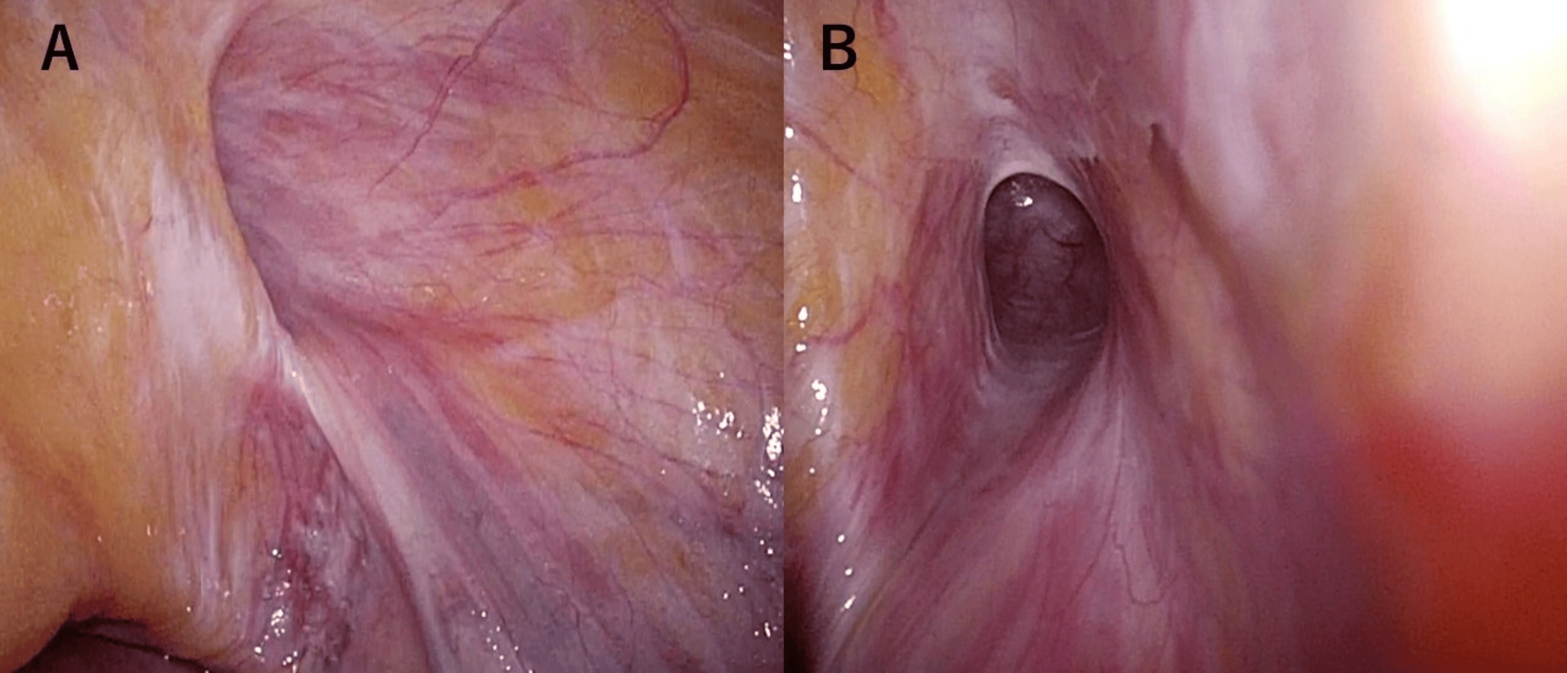
Intraoperative laparoscopic findings of the right inguinal region (A) Distant view of the right internal ring under low-pressure pneumoperitoneum (7 mmHg). (B) Close-up view confirming the entrance of the hernia sac continuing into the inguinal canal, consistent with a right indirect inguinal hernia (EHS classification L1). EHS: European Hernia Society

Before trocar removal, the pneumoperitoneum was reduced to approximately 7 mmHg for a final bilateral inspection. At this point, a subtle bulge was observed at the right internal ring, consistent with an indirect inguinal hernia (EHS classification L1) (Figure 3).

A right-sided TAPP procedure was therefore performed in the same session, with mesh placement and peritoneal closure similar to the left side.

The total operative time was 93 minutes, the estimated blood loss was negligible, and no intraoperative complications occurred. The postoperative course was uneventful. The patient resumed oral intake on the first postoperative day and was discharged according to the standard clinical pathway. At early follow-up, he reported minimal pain and no wound problems or recurrence.

## Discussion

Laparoscopic techniques such as TAPP and totally extraperitoneal repair allow comprehensive visualization of the myopectineal orifice and can reveal occult or concomitant hernias not apparent during open repair. Prior studies have demonstrated that 7-15% of patients undergoing TAPP harbor contralateral occult hernias [1-4]. International guidelines emphasize that laparo-endoscopic repair provides the opportunity to detect and repair these defects when encountered [2,5].

In our case, the contralateral hernia was not identified at the beginning of the procedure but was recognized during a final inspection before closure when the pneumoperitoneum was reduced. This suggests that the detectability of small hernias is not static but influenced by intraoperative conditions. One possible explanation is that sustained pneumoperitoneum over the course of the procedure gradually promoted protrusion of preperitoneal tissue through a weakened internal ring, thereby unmasking a latent defect that was initially inapparent. In addition, lowering the insufflation pressure may restore a more physiological abdominal wall tension, further contributing to the visualization of the defect. Thus, hernia detection during TAPP may be a dynamic phenomenon influenced by both the level and duration of pneumoperitoneum.

Recent reports also highlight that unexpected intraoperative findings during minimally invasive groin surgery may include non-inguinal groin hernias - most commonly femoral and, less frequently, obturator hernias - which often require strategies different from a straightforward indirect repair (e.g., different dissection planes and, when incarcerated, urgent management). Standardizing a brief second look before closure could therefore help reduce missed pathology not only for pressure-dependent occult inguinal defects but also for these non-inguinal entities [6-8].

These observations underscore two practical lessons for surgeons: (i) Inspection under different conditions of pneumoperitoneum - for example, at both standard and reduced pressures - may improve the likelihood of identifying pressure-dependent defects. (ii) Repeated inspections at multiple time points - at the start, during the procedure, and before closure - are essential, since a single survey may miss occult contralateral pathology.

Alius et al. have also proposed that the final step of TAPP should include a hemostasis check under standard pressure and an additional inspection at low pressure to reveal occult hernias [9]. Our case not only supports this concept but also suggests that time-dependent factors may play a role in unmasking contralateral hernias, highlighting the importance of structured, repeated assessments during the entire operation.

Therefore, we recommend that TAPP should incorporate both initial and final inspections, performed under different conditions such as low pneumoperitoneum and at different time points, to minimize the risk of missed contralateral hernias and ensure definitive repair in a single anesthetic session.

## Conclusions

Occult contralateral inguinal hernias may be inapparent at the start of TAPP under standard pneumoperitoneum yet revealed by a brief low-pressure inspection before closure, underscoring that detection is dynamic and condition-dependent. We therefore recommend a structured two-time-point inspection - an initial survey at standard pressure and a final re-survey at reduced pressure immediately before trocar removal.
